# Pancreatic Colloid Carcinoma Arising From Intraductal Papillary Mucinous Neoplasm in the Setting of Gallbladder Agenesis, Ansa Pancreatica, and Santorinicele: Follow-Up of a Previously Reported Case

**DOI:** 10.7759/cureus.104425

**Published:** 2026-02-27

**Authors:** Harine Siribaddana, Sun Woo Lee, Emily Shi, Fraser Simpson, Harsh Kandpal, Matthew Burge, Nicholas O'Rourke, Andrew Clouston, Manju D Chandrasegaram

**Affiliations:** 1 General Surgery, The Prince Charles Hospital, Brisbane, AUS; 2 Radiology, The Prince Charles Hospital, Brisbane, AUS; 3 Medical Oncology, The Prince Charles Hospital, Brisbane, AUS; 4 General Surgery, Royal Brisbane and Women's Hospital, Brisbane, AUS; 5 Pathology, Royal Brisbane and Women's Hospital, Brisbane, AUS

**Keywords:** ansa pancreatica, chronic pancreatitis, gallbladder agenesis, intraductal papillary mucinous neoplasia, pancreatic colloid carcinoma, pancreaticoduodenectomy, santorinicele

## Abstract

This report details the long-term follow-up of a patient previously reported from our centre with recurrent acute pancreatitis and episodic jaundice in the setting of three rare anatomic variants: gallbladder agenesis, ansa pancreatica, and a santorinicele. The index report described progressive main pancreatic duct dilatation and endoscopic findings consistent with intraductal papillary mucinous neoplasm (IPMN) despite non-diagnostic cross-sectional imaging. We now present the subsequent clinical course, including elective pancreaticoduodenectomy, definitive histopathology, and surveillance outcomes. Histopathology demonstrated a 30 mm grade 2 pancreatic colloid carcinoma arising in association with an intestinal-type IPMN involving both main and branch ducts (pT2N1). This occurred despite the absence of a discrete mass or guideline-defined high-risk features on cross-sectional imaging. The postoperative course was complicated, and later remnant duct dilatation raised concern for a late pancreaticojejunostomy stricture. The patient’s symptoms subsequently improved, and duct calibre decreased on surveillance imaging, with computed tomography (CT) imaging in February 2025 confirming no recurrence of malignancy. This longitudinal case demonstrates that guideline-negative cross-sectional imaging does not exclude invasive carcinoma in suspected IPMN, particularly when pancreatitis and ductal variants confound interpretation. It supports individualised management, multidisciplinary assessment, and timely consideration of resection in selected patients.

## Introduction

Intraductal papillary mucinous neoplasms (IPMNs) are mucin-secreting epithelial tumours of the pancreatic ductal system that may involve branch ducts, the main pancreatic duct (MPD), or both, and can progress from low-grade dysplasia to high-grade dysplasia and invasive carcinoma [1-3]. Main-duct IPMN (MD-IPMN) carries a higher likelihood of advanced dysplasia or invasive malignancy than isolated branch-duct disease [1-3]. Contemporary international guidance stratifies risk based on symptoms and imaging features, such as MPD calibre, cyst morphology, and mural nodules [1-3].

Pancreatitis can be associated with IPMN and is incorporated into diagnostic and escalation pathways, but recurrent inflammation can confound attribution of symptoms and ductal change [1-3]. In this context, cross-sectional imaging may remain indeterminate, and invasive disease may be present without a discrete mass or classic high-risk features [1-5]. When uncertainty persists, endoscopic evaluation may be required to better characterise intraductal pathology [1-3,6]. Peroral pancreatoscopy (SpyGlass) is an adjunctive endoscopic tool that can improve diagnostic confidence, support localisation of intraductal change, and influence management decisions [6].

Invasive carcinoma arising in association with IPMN includes distinct histologic subtypes, and colloid (mucinous) carcinoma is an uncommon invasive phenotype within this spectrum [4]. Imaging findings can be subtle or overlap with cystic and inflammatory change on computed tomography (CT) and magnetic resonance imaging (MRI), reducing conspicuity in some patients [4,5].

Here, we present an extended follow-up of a previously reported patient with recurrent pancreatitis and suspected MD-IPMN who was ultimately found to have invasive colloid carcinoma arising in association with IPMN at pancreaticoduodenectomy [7]. The case is notable for the coexistence of three uncommon anatomic variants: gallbladder agenesis, ansa pancreatica, and a santorinicele. These plausibly contributed to recurrent pancreatitis and obscured the attribution of symptoms and ductal change. This report highlights the challenge of malignancy risk assessment when cross-sectional imaging remains non-diagnostic over time and underscores the value of structured escalation, longitudinal multidisciplinary evaluation, and consideration of surgical management in complex phenotypes [1-3,6].

## Case presentation

A 33-year-old man first presented to the emergency department of our tertiary hospital in July 2017 with acute pancreatitis (lipase 3,670 U/L) and obstructive jaundice (bilirubin 95 μmol/L). He had previously been admitted four times for pancreatitis between October 2014 and June 2016. His medical history included gastro-oesophageal reflux disease (on pantoprazole), past smoking (quit in 2016 after 20 pack-years), minimal alcohol use, and no family history of pancreatic cancer.

The patient's early history to March 2020 is detailed in the prior case report [7]. He experienced recurrent mild acute pancreatitis (per the Revised Atlanta Criteria), with elevated liver function tests and bilirubin (Table 1) [8].

**Table 1:** Hepatobiliary and pancreatic enzymes during key pancreatitis episodes and follow-up (July 2017 to May 2023) GGT, gamma-glutamyl transferase; ALT, alanine aminotransferase; ALP, alkaline phosphatase; AST, aspartate aminotransferase. Reference ranges: bilirubin <20 μmol/L; GGT <55 U/L; ALT <45 U/L; ALP 30-110 U/L; AST <35 U/L; lipase <60 U/L. Episodes are labelled alphabetically in chronological order as described in the index case presentation. Units are shown in column headers. *Elevated above the reference range.

Time point	Bilirubin (µmol/L)	GGT (U/L)	ALT (U/L)	ALP (U/L)	AST (U/L)	Lipase (U/L)
Episode A (July 2017)	95*	309*	435*	244*	488*	3,670*
Episode E (November 2018)	32*	134*	130*	75	50*	406*
Episode F (December 2018)	57*	178*	203*	116*	136*	1,040*
Episode I (July 2019)	44*	147*	113*	72	74*	3,050*
Episode L (January 2020)	46*	248*	176*	90	216*	1,650*
Episode N (February 2020)	13	102*	41	89	26	3,850*
May 2023	14	62*	90*	81	36*	59

**Figure 1:**
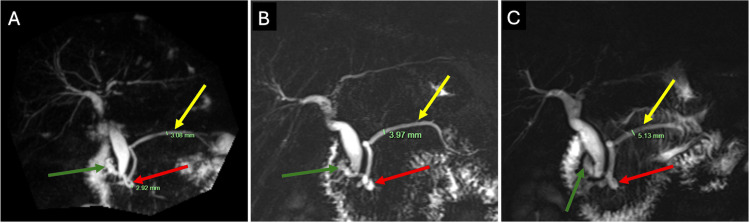
MRCP images demonstrating gradual progressive dilatation of the main pancreatic duct Coronal T2-weighted MRCP maximum-intensity projection images demonstrating progressive dilatation of the main pancreatic duct (yellow arrow), measuring approximately (A) 3 mm in November 2018, (B) 4 mm in September 2019, and (C) 5 mm in February 2020. A santorinicele (green arrow) and ansa pancreatica (red arrow) are also evident. MRCP, magnetic resonance cholangiopancreatography.

Cross-sectional imaging showed no peripancreatic collections, necrosis, or pancreatic masses. Rare anatomical variants were identified, including gallbladder agenesis (confirmed by nuclear medicine imaging), ansa pancreatica, and santorinicele. Serial magnetic resonance cholangiopancreatography (MRCP) demonstrated gradual MPD dilatation from 3 mm to 5 mm, with parallel enlargement of the ansa pancreatica (Figure 1).

**Figure 2:**
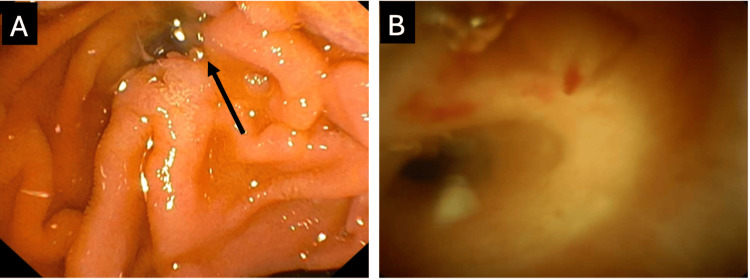
Endoscopic findings supporting main duct intraductal papillary mucinous neoplasm (March 2020) (A) ERCP photograph demonstrating mucin (arrow) extruding from the major papilla and (B) SpyGlass pancreatoscopy visualising the main pancreatic duct. ERCP, endoscopic retrograde cholangiopancreatography.

Endoscopic retrograde cholangiopancreatography (ERCP) in January 2018 demonstrated a patulous major papilla with mucin extrusion and a mobile intraductal filling defect. Endoscopic ultrasound (EUS) in February 2020 demonstrated echogenic material suspicious for mucin in the MPD, and mucinous expression from a prominent minor papilla. ERCP with SpyGlass pancreatoscopy (March 2020) visualised thick mucin and intraductal papillary projections in the pancreatic head and neck, supporting a diagnosis of MD-IPMN (Figure 2).

Apart from an isolated elevated cancer antigen 125 (CA 125) in 2017, his tumour markers remained unremarkable (Table 2).

**Table 2:** Serum tumour markers measured from July 2017 to May 2021 CA 19.9, carbohydrate antigen 19.9; CA 125, cancer antigen 125; CEA, carcinoembryonic antigen. Reference ranges: CA 19.9 <35 kU/L; CA 125 <35 kU/L; CEA <5.0 μg/L. “Not measured” indicates the test was not performed. *Elevated above the reference range, likely reflecting acute pancreatitis and obstructive jaundice.

Date	CA 19.9 (kU/L)	CA 125 (kU/L)	CEA (μg/L)
July 2017	28	321*	1.1
October 2017	6.5	18	1.6
May 2018	5.4	Not measured	1.4
March 2021	Not measured	12	Not measured
May 2021	16	Not measured	1.5

The case was reviewed in a hepatopancreatobiliary (HPB) multidisciplinary setting. Recurrent pancreatitis over approximately six years, progressive MPD dilatation from 3 mm (November 2018) to 5 mm (February 2020), and endoscopic evidence of intraductal mucin with papillary projections supported operative management. Although endoscopic mucin clearance improved symptoms, the patient continued to experience intermittent pain, requiring further hospital admissions. Given his young age, continued surveillance with repeat endoscopic management was considered, but definitive resection was ultimately recommended as the most durable strategy for symptom control and malignancy risk mitigation. Following discussion of risks and benefits and shared decision-making, the patient underwent elective open pancreaticoduodenectomy (Whipple procedure) in June 2020.

The operation was performed via a right subcostal incision and was technically demanding due to extensive chronic inflammatory change and dense fibrosis. The chronically inflamed pancreatic head was densely adherent to the transverse mesocolic vessels and superior mesenteric vein. Reconstruction included a duct-to-mucosa pancreaticojejunostomy (PJ) using the Blumgart technique. A hepaticojejunostomy (HJ) was formed, and gastrointestinal continuity was restored with a retrocolic, retrogastric gastrojejunostomy (GJ). Two intra-abdominal drains were placed, one posterior to the HJ and PJ and the second anterior to the GJ and PJ.

**Figure 3:**
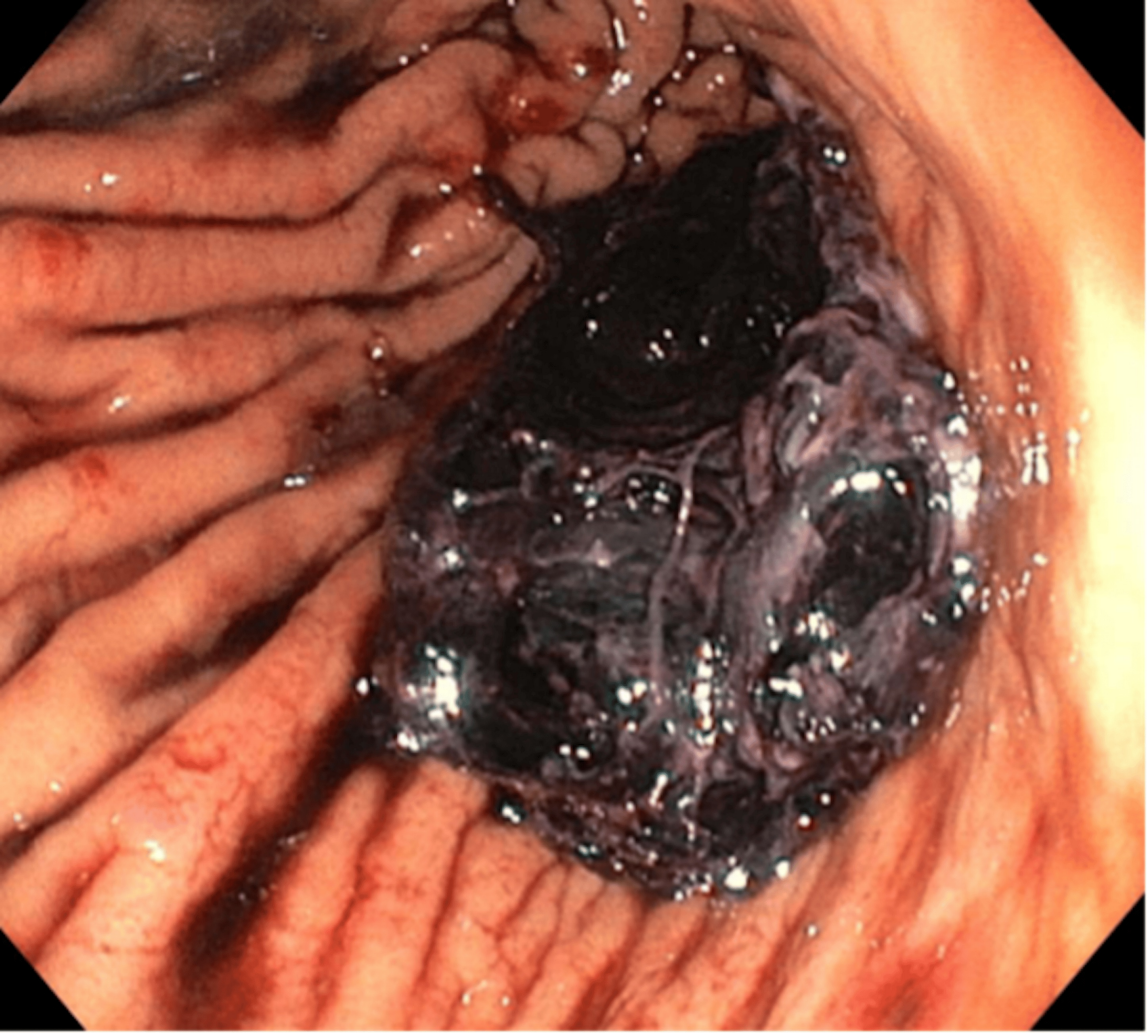
UGIE performed day 1 postoperatively for a suspected acute haemorrhage (June 2020) Endoscopic photograph demonstrating a clot over the gastrojejunostomy anastomosis. UGIE, upper gastrointestinal endoscopy.

Postoperative recovery was complicated by a suspected pancreatic fistula (drain fluid lipase of 3950 U/L), right pleural effusion, and intra-abdominal collections. On day 1 postoperatively, upper gastrointestinal endoscopy was performed for a suspected acute gastric haemorrhage. A large gastric clot (approximately 575 mL of haematin) was removed, and a clot over the GJ was carefully aspirated (Figure 3).

**Figure 4:**
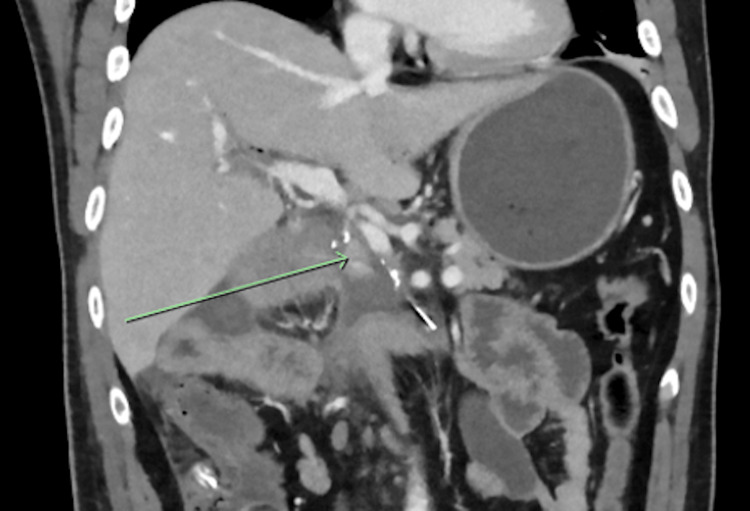
CT imaging day 8 postoperatively for a suspected acute intra-abdominal haemorrhage (June 2020) Coronal CT image with portal venous contrast enhancement demonstrating pooling of contrast (arrow) inferior to the gastroduodenal artery stump. CT, computed tomography.

On day 8, bleeding via the abdominal drain prompted CT imaging (Figure 4), which demonstrated acute haemorrhage from the gastroduodenal artery stump. This was managed with emergent image-guided embolisation.

**Figure 5:**
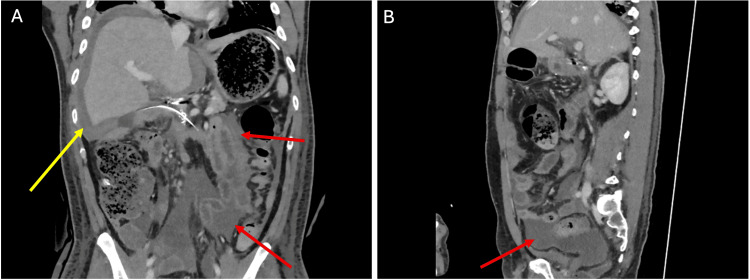
CT images day 18 postoperatively to investigate recurrent fevers and rising inflammatory markers (June 2020) CT imaging in (A) coronal and (B) sagittal views with portal venous contrast enhancement demonstrating intra-abdominal fluid (red arrows), predominantly surrounding the liver (yellow arrow). CT, computed tomography.

Ongoing haemodynamic instability led to relook surgery on day 13. A right subphrenic haematoma was washed out, and pus in the epigastrium with suspected communication to the PJ was aspirated. Recurrent fevers, a rising C-reactive protein (330 mg/L), and CT imaging confirming multiple intra-abdominal collections (Figure 5) led to a laparoscopic washout on day 18. Purulent fluid was aspirated, a subphrenic drain was placed, and a right intercostal drain was inserted to treat his persistent pleural effusion. Overall, four intensive care unit admissions were required during this hospitalisation, and he was discharged after a 31-day inpatient stay.

**Figure 6:**
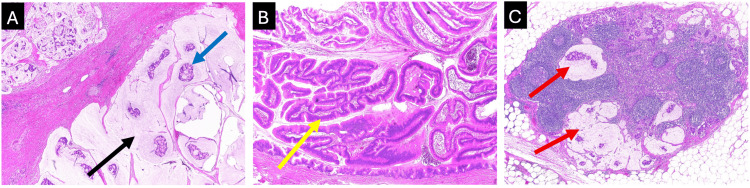
Histopathology from the pancreaticoduodenectomy specimen demonstrating pancreatic colloid carcinoma arising in association with an intraductal papillary mucinous neoplasm (June 2020) (A) Colloid (mucinous) adenocarcinoma showing abundant extracellular mucin (black arrow) with floating neoplastic epithelium (blue arrow). Haematoxylin and eosin (H&E) stain, original magnification ×20. (B) Associated intraductal papillary mucinous neoplasm with intestinal-type epithelium and papillary architecture (yellow arrow). H&E stain, original magnification ×40. (C) Metastatic carcinoma within a regional lymph node (red arrows). H&E stain, original magnification ×20.

Final pathology revealed a well-circumscribed solid cream lesion in the pancreatic head encircling the MPD. Histology confirmed a grade 2 colloid adenocarcinoma (Figure 6A) of the pancreas (pT2 pN1 by AJCC 8th edition criteria) arising in association with an IPMN [9]. The IPMN involved both main and branch ducts and was lined predominantly by intestinal-type epithelium with low-grade dysplasia (Figure 6B). The invasive colloid carcinoma measured 30 mm in maximum dimension and extended into the peripancreatic soft tissue and duodenal wall. Resection margins were negative for colloid carcinoma, and the pancreatic transection margin was also clear of IPMN. Lymphovascular and perineural invasion were not seen, and there was no neural plexus invasion at the superior mesenteric artery (SMA) margin. One of 12 lymph nodes was positive (Figure 6C). Mismatch repair protein expression was retained (MLH1, MSH2, MSH6, PMS2).

Following multidisciplinary team review, adjuvant chemotherapy was considered. The patient was reviewed by the medical oncology team postoperatively, and adjuvant chemotherapy was discussed in the context of pT2N1 disease (one out of 12 nodes positive) with negative margins and no lymphovascular or perineural invasion. In the setting of IPMN-associated colloid carcinoma, the limited evidence for benefit from adjuvant chemotherapy was weighed against his prolonged postoperative recovery with ongoing pain and deconditioning. A surveillance approach with close interval imaging was therefore chosen after shared decision-making, with chemotherapy to be reconsidered if surveillance revealed concerning findings.

**Figure 7:**
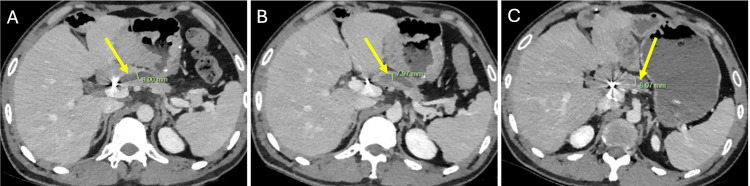
CT images demonstrating persistent dilatation of the remnant main pancreatic duct from March 2021 to April 2022 Axial CT imaging with portal venous phase contrast enhancement demonstrating persistent dilatation of the remnant main pancreatic duct (arrow) at approximately 8 mm in (A) March 2021, (B) October 2021, and (C) April 2022. CT, computed tomography.

From late 2020, the patient developed intermittent abdominal pain and was admitted for two days in November 2020 with mild pancreatitis (lipase 632 U/L). Imaging in March 2021 demonstrated dilatation of the remnant MPD to 8 mm with pancreatic atrophy, which persisted on CT in October 2021 and April 2022 (Figure 7).

**Figure 8:**
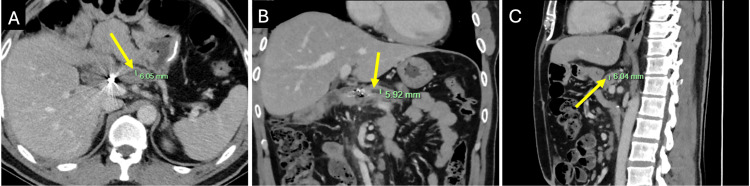
CT imaging in February 2025 demonstrated stable main pancreatic duct dilatation and no metastatic recurrence Portal venous phase contrast-enhanced CT images in (A) axial, (B) coronal, and (C) sagittal views demonstrating a 6 mm main pancreatic duct (arrow) on surveillance in February 2025. CT, computed tomography.

These findings, together with his symptoms, raised concern for a late PJ anastomotic stricture. Device-assisted double-balloon enteroscopy in June 2021 did not identify the PJ orifice or permit retrograde pancreatography of the PJ anastomosis. Further options were considered, including EUS-guided pancreatic duct access to facilitate rendezvous balloon-assisted ERCP. The patient’s symptoms subsequently improved, and, in view of his prior postoperative course, he elected to defer further intervention unless essential. His MPD calibre decreased to 6 mm on CT in May 2023 and remained stable on the most recent CT imaging in February 2025 (Figure 8).

On clinical review in February 2025, the patient reported sustained symptomatic improvement. His nutritional status improved with pancreatic enzyme replacement, and his weight increased and stabilised (body mass index of 27). CT imaging in February 2025 showed no recurrence of malignancy. Ongoing surveillance with CT imaging and clinical review is planned for early 2026.

## Discussion

This follow-up report describes the malignant transformation of an MD-IPMN to invasive colloid carcinoma. The patient was previously reported from our centre with recurrent pancreatitis, rare congenital pancreatic duct anomalies, and endoscopic features suspicious for IPMN [7]. Despite longitudinal progression, cross-sectional imaging (CT and MRCP) and endoscopic assessment (ERCP and EUS) did not identify a discrete mass or demonstrate guideline-defined high-risk stigmata. This case, therefore, illustrates the limitations of relying on static imaging thresholds in complex pancreatitis phenotypes. In our patient, the decision to proceed with pancreaticoduodenectomy was driven by a trajectory-based multidisciplinary evaluation rather than a single imaging threshold. This integrated recurrent pancreatitis, progressive MPD dilatation (Figure 1), and intraductal findings of mucin with papillary projections on pancreatoscopy, supporting definitive management despite equivocal imaging [1-3,6].

Several anatomical variants plausibly contributed to recurrent pancreatitis and confounded the attribution of symptoms and duct dilatation. Gallbladder agenesis is rare (reported population incidence of approximately 0.01% to 0.075%), often misinterpreted on ultrasonography, and typically requires cross-sectional imaging for confirmation [10]. Ansa pancreatica is an uncommon ductal configuration involving a looping communication between the ventral and dorsal pancreatic ducts [11,12]. It is identified in around 1% of MRCP examinations and has been associated with recurrent acute pancreatitis, plausibly through altered flow dynamics and impaired ductal drainage [11,12]. Santorinicele, a focal cystic dilatation of the terminal dorsal duct near the minor papilla, may further contribute to dorsal duct outflow obstruction [13]. Santorinicele without pancreas divisum (as in our patient) is rarely reported [13,14]. Collectively, these anomalies can be considered a plausible alternative explanation for gradual MPD enlargement. However, persistent symptom burden, progressive MPD dilatation, and subsequent endoscopic intraductal findings ultimately favoured evolving MD-IPMN [7].

IPMN, particularly main-duct and high-risk phenotypes, carry a recognised risk of high-grade dysplasia and invasive malignancy [2,3,15]. Contemporary guidance (Kyoto, revised Fukuoka, and European evidence-based recommendations) uses high-risk stigmata and worrisome features to direct surveillance, intensified assessment, or resection [1-3]. The patient’s features are mapped against commonly used criteria in Table 3.

**Table 3:** Key patient findings mapped to selected high-risk and worrisome criteria across intraductal papillary mucinous neoplasm guidelines MPD, main pancreatic duct; IPMN, intraductal papillary mucinous neoplasm; CA 19-9, cancer antigen 19-9. Fukuoka 2017 terms: “high-risk” indicates high-risk stigmata and “worrisome” worrisome features [2]. European 2018 terms: “absolute” indicates an absolute indication for surgery and “relative” a relative indication [3]. Italian 2014 terms: “treatment” indicates an indication for treatment and “suspicious” a suspicious feature [16]. Selected criteria relevant to escalation or resection are shown. “Not applicable” indicates no definable pancreatic cyst.

Criterion	Patient finding	Fukuoka 2017	European 2018	Italian 2014
MPD ≥10 mm	Absent (MPD 5-6 mm)	High-risk	Absolute	Treatment
MPD 5-9 mm	Present (MPD 5-6 mm)	Worrisome	Relative	Suspicious
Obstructive jaundice attributable to cystic lesion or tumour	Not clearly IPMN-related	High-risk when associated with a pancreatic head cystic lesion	Absolute (when tumour-related)	Suspicious
Enhancing mural nodule or solid component	Absent	High-risk if ≥5 mm, worrisome if <5 mm	Absolute	Treatment if enhancing solid component
Cyst features (size, wall, growth)	Not applicable (no pancreatic cyst)	Worrisome if size ≥3 cm, thick wall, or growth >5 mm per 2 years	Relative if size ≥40 mm or growth ≥5 mm per year	Suspicious if size ≥3 cm or thick wall
Elevated serum CA 19-9	Absent	Worrisome	Relative in the absence of jaundice	Suspicious
Abrupt duct change with distal atrophy or lymph nodes	Absent	Worrisome	Not specified	Suspicious (abrupt change)
Acute pancreatitis attributable to IPMN	Present (recurrent pancreatitis)	Worrisome	Relative	Symptom prompting escalation
New-onset diabetes mellitus	Absent	Not specified	Relative	Symptom prompting escalation
Positive cytology (high-grade dysplasia or malignancy)	Not obtained	Not specified	Absolute	Not specified

In this patient, the MPD calibre reached 6 mm, and an enhancing mural nodule or cystic structure was not identified. Therefore, definitive high-risk criteria for upfront surgery (such as an MPD of 10 mm or greater) were not met. However, the patient’s MPD dilatation in the 5 to 9 mm range and recurrent pancreatitis are considered worrisome features in the Fukuoka framework, with MPD calibre similarly emphasised in the Italian consensus [2,16]. Accordingly, these findings prompted escalation of assessment rather than reassurance. This context highlights a central interpretive challenge: pancreatitis with duct dilatation may reflect either IPMN-related obstruction or chronic pancreatitis with secondary ductal change. Distinguishing between these entities is critical to operative decision-making [2].

In this setting, pancreatoscopy represents a pragmatic escalation step in symptomatic patients with progressive ductal change and discordant cross-sectional imaging, informing management when guideline thresholds are not met [1-3,6]. ERCP identification of mucin at the papilla, together with SpyGlass pancreatoscopy demonstrating intraductal mucin and papillary projections, is strongly supportive of MD-IPMN when interpreted within the appropriate clinical context [7]. A recent systematic review and meta-analysis support peroral pancreatoscopy as an adjunct in the diagnostic work-up of IPMN [6]. However, technical limitations are recognised, including ductal angulation and incomplete assessment of upstream segments, both of which were relevant in this patient [6].

This case also highlights that invasive colloid carcinoma may be radiologically occult on CT and MRI, particularly in the setting of recurrent pancreatitis. Colloid carcinoma is characterised by abundant extracellular mucin and may appear cyst-like or infiltrative with relatively low enhancement compared with tubular adenocarcinoma [4,5]. On MRI, lesions are often markedly T2 hyperintense with minimal or delayed enhancement, whereas CT may show ill-defined low attenuation that is difficult to distinguish from pancreatitis-related fluid, oedema, or fibrosis [4,5]. Invasive carcinomas arising in association with IPMN may therefore be less conspicuous when colloid histology is present, increasing the risk of guideline-negative imaging despite invasive disease [4]. Consistent with this, serial MRCP in our patient (Figure 1) demonstrated progressive MPD dilatation without a discrete mass, despite a 30 mm colloid carcinoma on final pathology.

Colloid carcinoma arising in association with IPMN is uncommon and is often characterised by a more indolent course than conventional pancreatic ductal adenocarcinoma and tubular invasive carcinoma arising from IPMN, although nodal metastasis remains prognostically adverse [17,18]. Evidence supporting adjuvant chemotherapy after resection of invasive IPMN remains limited and heterogenous, and colloid tumours are underrepresented in retrospective series [18-21]. While selected cohorts and pooled analyses report an association between adjuvant chemotherapy and improved survival in higher-risk pathology, subtype-specific inference for colloid histology is constrained by small numbers and selection bias [18,21]. In contrast, the international multicentre ADENO-IPMN study of 459 patients found no association between adjuvant chemotherapy and recurrence or survival, including in high-risk subgroups such as lymph node-positive disease [19]. A multicentre study of IPMN-derived pancreatic cancer suggested that any survival association with adjuvant chemotherapy may be concentrated in patients with nodal disease and elevated CA 19-9, reinforcing the need for individualised decision-making rather than routine treatment for all patients [22]. Accordingly, we individualised management in our patient with pT2N1 disease (one out of 12 nodes positive) but favourable pathological features such as clear margins without lymphovascular or perineural invasion. Adjuvant chemotherapy was deferred after shared decision-making, given the uncertain incremental benefit in IPMN-associated colloid carcinoma and the patient's prolonged postoperative morbidity, with close surveillance adopted and chemotherapy to be reconsidered if concerning features emerged.

Finally, this extended follow-up highlights PJ anastomotic stricture as an important benign cause of recurrent pancreatitis after pancreaticoduodenectomy. Such strictures may present years after surgery with pancreatic-type pain, remnant MPD dilatation, and recurrent pancreatitis [23]. Management is challenging in altered anatomy and may include device-assisted ERCP, EUS-guided pancreatic duct drainage with antegrade stenting or rendezvous techniques, and surgical revision in selected cases [23].

Overall, this report illustrates that invasive disease may be present in suspected MD-IPMN without a discrete mass or guideline-defined high-risk stigmata on cross-sectional imaging, particularly when pancreatitis and anatomic variants confound interpretation. In our patient, worrisome features, including recurrent pancreatitis and MPD dilatation in the 5 to 9 mm range, justified escalation within existing guideline frameworks rather than reassurance. A longitudinal, multidisciplinary approach integrating symptom evolution, duct calibre trajectory, and intraductal endoscopic findings can reduce diagnostic uncertainty and support timely definitive management in selected patients. Given the need for advanced endoscopy, complex HPB surgery, interventional radiology, and intensive care support, complex IPMN phenotypes like this are best managed in high-volume tertiary referral centres with full multidisciplinary expertise.

## Conclusions

This report illustrates that progressive MD-IPMN with invasive colloid carcinoma can develop in a patient whose recurrent pancreatitis is initially attributed to rare congenital biliary and pancreatic duct anomalies. Invasive colloid carcinoma may remain occult on cross-sectional imaging, and guideline imaging thresholds may not capture all patients at risk in complex phenotypes, particularly when pancreatitis obscures subtle invasion.

When the symptom trajectory is severe or escalating, and intraductal endoscopic findings demonstrate mucin and papillary change, timely definitive management should be considered even when CT, MRI, and EUS do not show a discrete mass.
